# Purveyors of the Commercial Determinants of Health Have No Place at Any Policy Table Comment on "Towards Preventing and Managing Conflict of Interest in Nutrition Policy? An Analysis of Submissions to a Consultation on a Draft WHO Tool"

**DOI:** 10.34172/ijhpm.2020.171

**Published:** 2020-09-06

**Authors:** Ronald Labonté

**Affiliations:** School of Epidemiology and Public Health, University of Ottawa, Ottawa, ON, Canada.

**Keywords:** Conflict of Interest, Public Health Protection, Public Goods

## Abstract

With public health attention on the commercial determinants of health showing little sign of abatement, how to manage conflicts of interest (COI) in regulatory policy discussions with corporate actors responsible for these determinants is gaining critical traction. The contribution by Ralston et al explores how COI management has itself become a terrain of contestation in their analysis of submissions on a draft World Health Organization (WHO) tool to manage COI conflicts in development of nutrition policy. The authors identify two camps in conflict with one another: a corporate side emphasizing their individual good intents and contributions, and an non-governmental organization (NGO) side maintaining inherent structural conflicts that require careful proscribing. The study concludes that the draft tool does a reasonable job in ensuring COI are avoided and policy development sheltered from corporate self-interests, introducing novel improvements in global governance for health. At the same time, the tool appears to adhere to a belief that private economic (corporate) and public good (citizen) conflicts can indeed be managed. I question this assumption and posit that public health needs to be much bolder in its critique of the nature of power, influence, and self-interests that pervade and risk dominating our stakeholder models of global governance.

## Introduction

 Much is being written on the ‘commercial determinants of health;’ since 2016 Google Scholar indicates more than 133 000 items. The focus is generally on a few ‘unhealthy commodities’ (tobacco, alcohol, sugar, ultra-processed foods), with less attention given to the deep capitalist and neoliberally incentivized logic of profit maximization. A separate stream of public health inquiry concerns itself with the environmental damages and health inequities arising from fossil fuel and mineral extractions, often emphasizing the harms caused to Indigenous peoples. If one also considers the egregious inequities arising from speculative finance, tax avoidance/evasion, and the widespread deregulation of liberalized global capitalism, there are multiple economic practices that might be considered ‘commercial determinants of health.’ As such, there are layers that could be peeled back when, in our embrace of global public-private partnerships, we confront the vexatious issue of protecting against conflicts of interest (COI).

The contribution by Ralston et al^1^ wade headlong into this political briar patch with their study of how different actors believe COI should be managed in the development of nutrition policy. Their case is the World Health Organization’s (WHO’s) online consultation on a 2017 draft tool intended to assist member states in managing COIs in developing their food-related policies. The authors adopt an approach increasingly popular in critical public health research in which texts are thematically analyzed to identify how specific policy problems are framed, and how such framing arises from, and preconditions towards, certain *a priori *policy choices. This approach has been usefully applied in studies of global health diplomacy (how health is framed as a foreign policy concern),^2^ trade and investment treaties,^3^ and even to differing conceptualizations of ‘global health.’^4^ Some studies adopt the heuristic developed by Bacchi,^5^ better known as WPR, or ‘what’s the problem represented to be,’ a six-question process to unpack how policy problems are implicitly framed. Ralston et al^1^ offer a more stripped-down model, content with interrogating their data base of 44 submissions to identify two key policy frames (collaboration and partnership; and conflict and restricted engagement), each with different problem definitions (representations) and explicit or implicit policy solutions. Despite the acknowledged limitation of the data base, the findings delineate a largely bifurcated universe in conflict with itself: On the one side there is a private (corporate) sector and the United States (alone of the six member states making submissions) arguing that the draft COI guidelines are unnecessary, unfair, and contradictory to the United Nations (UN) and Sustainable Development Goal pivots towards increased public-private partnerships. On the other side are the non-governmental organizations (NGOs), most academic institutions (in itself, a noteworthy finding not commented upon by the authors), and the remaining five member states, either supporting the important novelty of the new tool or, in a few NGO instances, criticizing it for not going far enough in preventing the ‘foxes’ from ‘guarding the chicken coop’ (a colourful phrase underscoring the risks of regulatory capture).

## Individual or Institutional COI?

 The draft WHO tool distinguishes between two types of interests: individual, which seems to apply to personal gain or ideological commitments in conflict with a government policy goal; and institutional, which emphasizes more structurally embedded ‘economic, commercial, or financial’ interests that may be in conflict with public health policy. The authors’ textual analysis suggests that corporate sector submissions present individualized arguments against the draft tool, viz. that individual firms (or their trade associations) already have sufficient transparency with their voluntary codes of conduct, thus making additional measures to estimate potential conflicts unnecessary. NGOs take the opposite stance, arguing that, collectively, the commercial food industry is in structural (institutional) conflict that needs careful and ongoing interrogation as (partly) afforded by the new tool. Several additionally posited that private economic interests and the push for ‘multi-stakeholder governance’ and ‘public-private partnerships,’ with their embedded COI assumptions, is putting at risk the protection of public interests. One NGO submission is cited as noting that the draft WHO guidelines continues to “blur the line between public and private,” notable for surfacing the inherent bias in all COI constructs: that differing interests appear to be given equivalent moral weight and, as several of the corporate submissions contend, are little more than (slightly) differing opinions easily managed in respectful dialogue. Their interests are no different from anyone’s (or any institution’s) interests; they are just interests. An almost whining complaint found in some of the corporate submissions is with the “tone” of the WHO draft tool; unlike the florid language of partnerships in other UN documents, the draft tool is seen as non-inclusive and unwelcoming of (or even “hostile to”) any private sector participation. Their rejoinder to this “unfair” perception is to declare that “commercial motives are not incompatible with public health interests.” This may be an idealized possibility, but it is one belied by over a century of documented and often court-attested corporate malfeasances across multiple economic sectors with impacts directly or indirectly “hostile” to public health.

## Public Interests Are not the Same as Private Interests

 This conflation of public and private interests noted by some of the submissions the authors examined is the most troubling aspect of what they describe as “the high levels of contestation surrounding the very concept of COI.” With (perhaps admirable) academic neutrality, their discussion of this point does not probe deeply on the nature of this contestation, but simply recapitulates the essence of the two sides’ arguments. Their own position with respect to the respective merits of the two sides’ arguments in contest (conflict?) with each other remains unstated. One might read between the lines, or any of their other contributions on the topic of the globalization of unhealthy food commodities, to infer a tilt in the direction of the NGO angels of public interest. Still, the article’s conclusion carries this notion of interest equanimity further, affirming the potential of the draft tool “to move past a blanket acceptance or rejection of partnership to identify specific actors and forms of engagement where COI can be managed in ways that protect public health nutrition goals.” They judge the tool to be an important innovation in global governance for health, at least within the nutrition policy space.

 This may be true. And the tool, at least as based on its synopsis by the authors, does appear to offer improvements in the stringencies with which private (corporate) interests should be assessed before their representatives are invited to sit around the regulatory policy table. What remains unsaid, however, is that the economic and political power of those private interests massively outweigh those of groups arguing for the public good. The crudity of such power imbalances are increasingly on view as science and reason are easily dismissed as the ‘fake news’ of ‘elites,’ assiduous fact-checking fails to quell the proliferation of lies or conspiracy theories, and civil society protest in the name of public interest is increasingly met with militarized repression. Such issues go beyond the remit of the article’s intent. Moreover, the article, and the case study it draws from, pre-date the coronavirus disease 2019 (COVID-19) era. The status quo disruption caused by the pandemic has many academic think tanks and civil society organizations trying to envision and promote a post-COVID-19 ‘normal’ bearing scant resemblance to the one that many governments still seem committed to recreating.

## Conclusion

 The authors’ final words in the article identify “a pressing need for the development of a more detailed typology of COI that can be operationalised and applied in diverse policy contexts.” There is little argument there.

 But I would append to this the greater need for a differing conceptualization of COI (one that clearly locates conflicts as inherent within capitalist market systems, however successfully their neoliberal excesses might become throttled) and detailed policy playbooks that begin to address our (still existential) crises of global wealth/power inequalities, climate change, environmental overshoot, and gross imbalances in the excessive consumption by some to the health-damaging under-consumption by others. COI around nutrition policy is simply one small eddy in a much greater geopolitical turbulence.

## Ethical issues

 Not applicable.

## Competing interests

 Author declares that he has no competing interests.

## Author’s contribution

 RL is the single author of the paper.
